# Analysis of Genetic Diversity in the Czech Spotted Dog

**DOI:** 10.3390/ani10081416

**Published:** 2020-08-14

**Authors:** Karolína Machová, Anita Kranjčevičová, Luboš Vostrý, Emil Krupa

**Affiliations:** 1 Department of Genetics and Breeding, Faculty of Agrobiology, Food and Natural Sciences, Czech University of Life Sciences, 165 00 Prague, Czech Republic; kranjcevicova@af.czu.cz (A.K.); vostry@af.czu.cz (L.V.); 2 Department of Genetics and Breeding of Farm Animals, Institute of Animal Science, 104 01 Prague, Czech Republic; krupa.emil@vuzv.cz

**Keywords:** genetic variability, pedigree analysis, small population, inbreeding

## Abstract

**Simple Summary:**

Losses of genetic diversity have a particular impact on breed populations in countries with a small breeding base or national breeds with a small registry. Among them, it is possible to include the breed of the Czech Spotted Dog (CSD), whose variability has been low since the beginning of breeding due to the small number of founders and mating of close relatives. Through study of its pedigree records, we recorded a severe loss of genetic variability and high relatedness between animals. Moreover, the population is not free of genetic diseases; therefore, future existence of the breed is in danger.

**Abstract:**

Loss off genetic diversity negatively affects most of the modern dog breeds. However, no breed created strictly for laboratory purposes has been analyzed so far. In this paper, we sought to explore by pedigree analysis exactly such a breed—the Czech Spotted Dog (CSD). The pedigree contained a total of 2010 individuals registered since the second half of the 20th century. Parameters such as the mean average relatedness, coefficient of inbreeding, effective population size, effective number of founders, ancestors and founder genomes and loss of genetic diversity—which was calculated based on the reference population and pedigree completeness—were used to assess genetic variability. Compared to the founding population, the reference population lost 38.2% of its genetic diversity, of which 26% is due to random genetic drift and 12.2% is due to the uneven contribution of the founders. The reference population is highly inbred and related. The average inbreeding coefficient is 36.45%, and the mean average relatedness is 74.83%. The effective population size calculated based on the increase of inbreeding coefficient is 10.28. Thus, the Czech Spotted Dog suffered significant losses of genetic diversity that threaten its future existence.

## 1. Introduction

Many dog breeds suffer from limited genetic diversity. Due to their current use as human companions, they are selected by humans—and not by nature—for specific phenotypes. The strict division into breeds and forms causes irreversible loss of alleles and, in most cases, vitality as well. The bottleneck effect, use of favored sires, inbreeding and genetic drift are the main mechanisms of the loss of genetic diversity.

Genetic drift is a significant evolutionary force that is the functional essence of all of the other causes of the loss of genetic variability listed above [1]. The bottleneck effect occurs by selecting a low number of animals for breeding or subdividing a population. The current dispersion of dog hobby breeding all over the world creates a new form of the bottleneck effect—the national bottleneck [2]. This phenomenon occurs after the introduction of a breed into a new country. National breeds that have recently gained international recognition are the most affected [2]. Breeders are not able to maintain the same heterogeneity in newly established subpopulations as in the initial population, despite efforts to exchange genetic material internationally. Thus, heterozygosity decreases with subsequent generations and inbreeding increases [3]. However, subpopulations are also present within one studbook. For example, for poodles, four sizes (standard, medium, miniature and toy), five full coat colors (black, white, brown, gray and fawn) and two types of fur (curly and corded) are recognized by the breeding standard [4]. These varieties form separate subpopulations, among which gene flow is an undesirable phenomenon. Research on poodles registered in the Swedish Kennel Club revealed a division into a total of five subgroups based on a combination of size and color. The standard poodle group is even genetically distant from smaller subsets of instincts from another pure-bred breed, based on paired fixation index (F*_ST_*) measurements [5].

The negative consequences of current breeding strategies are becoming increasingly apparent in terms of population health status, and the welfare of individuals is starting to be discussed [6]. In the previous ten years, the genetic variability of many breeds has been analyzed across countries. Some of them are complex [7,8,9], and some of them are exclusively of a genealogical [10,11,12,13,14,15] or molecular nature [16,17]. However, all of them showed some disconcerting data in at least one studied breed. Rare and national breeds such as Polish Hounds [18] or Ca mè [19] are usually the most affected by the loss of genetic diversity. This is also the case for the Czech Spotted Dog (CSD). The breed was bred in the 1950s by František Horák for laboratory purposes. Breeding was aimed at the creation of a dog with a calm, mild nature and high fertility that was easy to care for, with a suitable body structure, size and coat. The founding individuals were two hybrids of unknown origin with the desired phenotype. Several other individuals were later crossed with them. However, breeding was (mainly from the beginning) largely based on inbreeding. The result was a medium-sized tricolor spotted breed with tilted ears with a lively, friendly nature. However, some genetically based diseases are relatively abundant in the CSD. The most common are hip dysplasia with a prevalence of 63.58%, patella luxation occurring in 53.44% of individuals or dental abnormalities in almost every seventh individual [20]. For this reason, we were interested in what the genetic diversity of the breed looks like now, after more than 60 years of development of the breed. The aim of our study was (a) to carry out a genealogical investigation of the Czech Spotted Dog, (b) to analyze its genetic diversity, (c) to evaluate current breeding practices and (d) identify possible strategies for the future.

## 2. Materials and Methods

Pedigree data were obtained from the KCHMPP kennel club (Klub Chovatelů Málopočetných Plemen Psů O.S., Vrdy, Czech Republic), which associates breeders of rare breeds. The data contained 2010 registered dogs from 4 August 1954 until 28 September 2017 with a record of their sex, kennel and names of parents. In 32 dogs, there was no information about birthdate or year of birth. A reference population (RP) for the analyses was defined as the population of interest that includes living and possibly reproducing animals. These animals were gained from lists of stud sires and dams as of 31 December 2017 available on the official KCHMPP website [21,22]. The RP consists of 234 individuals, 114 sires and 120 dams.

First, the completeness of the pedigree was computed by three methods: the maximum number of generations traced, the equivalent complete generations (ECG) and the pedigree completeness index (PCI). Because of the relatively small size of the pedigree, these completeness values should provide better insight into other computed parameters. ECG was obtained by the arithmetical mean of the sums of expected ancestors genetic gains over generations towards a given individual from the reference population [23].

For PCI calculation, a method based on the expected genetic contribution of paternal and maternal lineages was used that was similar to that used in the studies of Sørensen et al. [24] or Voges and Distl [25]. In this study, the five previous generations were used for the calculations (g = 5). For overall assessment within the pedigree, the arithmetic mean was calculated from the individual’s PCI either in the registration year or in the reference population, similar to the ECG. If the origin of only one line (maternal/paternal) of the ancestors is known, inbreeding cannot be detected, and the PCI is zero [26].

Identification of individuals overused in breeding was performed using the methodology of management of breeding CSD [27], which recommends not to exceed 5% of the total number of puppies registered in the last five years for each animal involved in breeding.

The reference population of 114 sires and 120 dams was used to compute the probability of gene origin. The total and the effective number of founders and ancestors and the founder genome equivalent were calculated. Subsequently, founders and ancestors with the highest genetic contribution to the reference population were identified. The effective number of founders (*f_e_*) and ancestors (*f_a_*) was calculated based on the method established by Lacy [28] and adjusted according to Boichard et al. [23].

The effective founder genome count (*f_ge_*) was determined as half the inverse of the average coancestry of the reference population [29].

The loss of genetic diversity based on genetic drift and bottleneck effect (1-GD) was calculated by Lacy [30]. The loss of genetic diversity based on the uneven contribution of the founders was computed according to Caballero and Toro [29]. Finally, the variability loss by genetic drift was found through simple subtraction of the GD values from the GD*.

The individual inbreeding coefficient was calculated using an algorithm developed by Meuwissen and Luo [31]. Additionally, its average values over the years were determined according to the date of registration of individuals in the pedigree (1989–1991; 1993–2017). In 1992, there were no registrations. In addition, individuals with unknown birth dates (registration) were assigned to six time periods (?–1954; 1955–1960; 1961–1978; 1979–1983; 1984–1985; 1986–1987) according to their registration number. These periods were chosen to begin and end with a year containing animals with known dates of birth and span those with unknowns.

The inbreeding coefficient (F) corrected for the PCI value was obtained from the study [25] as the F/PCI. The average PCI at different time points was calculated for individuals based on into the same periods as those used for the calculation of F except for the periods ?–1954 and 1955–1960 and the years 1990, 1991 and 1993, when the calculation was meaningless due to incomplete ancestral data. The increase in the coefficient between two generations in the total population was also determined [32].

The effective population size *N_e_* for the reference population was calculated as half of the average increase in individual inbreeding coefficients in the reference population [32,33].

The average relatedness (AR) was obtained based on the affinity matrix of *n* × *n* individuals, where the line vector *c*’ containing the AR coefficients of the individuals expresses the relationship described by Dunner et al. [34].

Most indicators were determined using Endog version 4.8 [35].

## 3. Results

### 3.1. Basic Demographic Data and Completeness of the Pedigree

Since the establishment of the pedigree at the end of 2017, a total of 949 female and 1061 male dogs have been registered. From 1954 to 1997, only 93 individuals were registered. Since 1998, the average number of registrations per year has been 93.9. Per sire (of total 141), there is an average of 14.42 puppies and per dam (189); the average is 10.58 puppies. The development of basic demographic parameters over time is shown in Table 1. In 2001–2017, 48 individuals were identified, including 24 dams and 24 dogs, as overexploited animals in breeding. Table 2 shows the overuse of specific individuals in specific years.

Ancestors of the reference population could be traced up to a maximum of 20 generations; the average value of complete equivalent generations for the reference population was 10.07 and the pedigree completeness index was 0.82. In the whole population, it was possible to trace up to 21 generations. The average of the complete equivalent generations of the whole group of individuals reached almost 9.46 generations, and the average of the pedigree completeness index to five generations reached back 78%. The average PCI was generally the lowest at the beginning of the breeding. In the years 1961–1978, it was 0.54, followed by an increase to one during 1979–1987, but only in a very small number of individuals (13 registrations in total). A further decline followed when the completeness of the pedigree was either very low or not measurable at all. Since 1995, it has reached stable values above 0.6, and since 2008 it has steadily increased from 0.65 to 0.96 in 2017. The completeness index of the reference population was also relatively high at 0.82.

### 3.2. Contribution of Ancestors and Founders to the Reference Population and Loss of Genetic Diversity

The basic population of the breed consisted of only ten individuals (five females and five dogs), of which nine founders (without one female) genetically participated in the creation of the current reference population. The effective number of founders (*f_e_*) was four, and the effective number of ancestors (*f_a_*) was three. Comparison of the values of *f_a_* and *f_e_* shows only a minimal effect of the bottleneck—*f_a_/f_e_* = 0.75. The founder genome equivalent of the reference population was 1.31. When comparing this value with the total number of founders, it was found that only 14.6% of the genetic variability of the base population reached the current reference population, and 85.4% was lost over generations.

To explain 50% of the genetic contribution to the reference population, only two ancestors would be needed, and only five ancestors would participate in more than 90% of the whole genetic diversity of the reference population. The biggest contributor was dam Dáša 6 (reg. num. 9) with 48.08%, Peggy (reg. num. 260) with 23.94% and the sire Míša (reg. num. 1) with 12.02%. The remaining ancestors were involved in less than 10%.

GD was found to be very high, 38.2%. GD*, explained by the uneven contribution of the founders, accounts for 12.2% of that, and genetic drift losses account for approximately 26% of the total GD losses.

### 3.3. Inbreeding Coefficient and Average Relatedness

The mean value of F and AR for the whole population were 35.61% and 75.94%, respectively. For the reference population, the values were 36.45% and 74.83%, respectively. Table 3 provides a comparison of the average AR and average inbreeding coefficient in each generation with the percentage of inbred individuals (with nonzero F). The numbers of inbred individuals in the reference population are compared with the total number of all registered individuals in Table 4. Most of the breeding animals have an inbreeding coefficient between 0.50 and 0.60.

**Figure 1:**
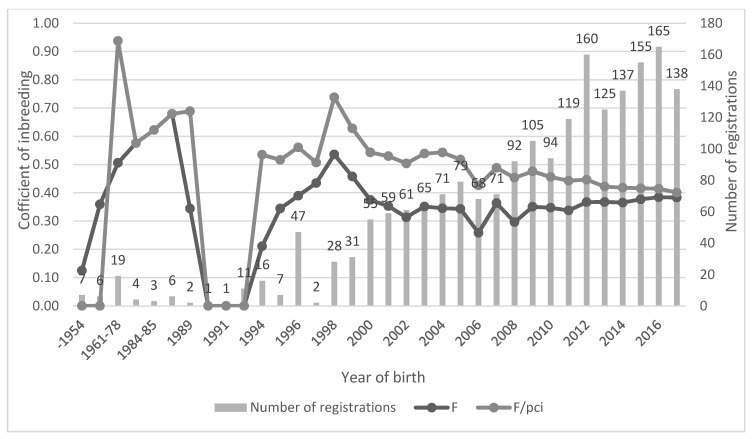
Coefficient of inbreeding, correction of inbreeding coefficient to pedigree completeness index (PCI) and number of registrations. Years without any registration were excluded (1988 and 1992).

An average coefficient of inbreeding for the registration period is shown in Figure 1 together with the number of registered animals in a given period. In Figure 1 there are also F values shown as corrections to PCI. Until 1987, the highest increase of F was up to 0.67 (PCI correlated with F was the same). The next period was very poor on registrations as well as knowledge of ancestors that resulted in a decrease until 1993 to zero. From 1994 to 1998, F increased again to 0.53. After the year 1999, a new decrease started and ended on a final value of 0.38 (in 2017). Along with this last decrease of the coefficient of inbreeding, F values became closer to the corrected PCI values to less than two standard deviations from the average of PCI corrected F (2*σ* = 0.2138) and, until 2011, were even less than one standard deviation (*σ* = 0.1076).

The increase in inbreeding (ΔF) in the total population was 0.82% per generation for the maximum number of generations and 3.48% per generation for the equivalent number of complete generations. The effective population size for the reference population, calculated based on an individual increase in inbreeding to the equivalent number of complete generations, was 10.28.

## 4. Discussion

This study aimed at examining a dog breed that is quite young. Although the generation of its founders occurred in the 1950s, its real creation (or recreation) started after 1990. This is the reason for its very small population (2010 registered individuals as of 2017) despite there being quite a high number of maximum generations (21). According to Shariflou et al. [36] and the distribution by the registry size, the CSD registry falls into the medium registry category (2001–25,000 records). The pedigree completeness for individuals from the reference population based on the equivalent complete generations (10.07) is very high compared to that of other breeds in the same category of the registry. For example, in Belgium, the value of ECG of medium registry breeds ranges between 3.4 and 7.1 [8], whereas for breeds registered in France it ranges between 2.9 and 6.9 [37] and for breeds in Australia it is 8.5 on average [36]. Furthermore, the knowledge of the ancestors of the reference population for the last five generations is almost complete. This is mostly due to the national character of the CSD breed. FCI has not recognized it yet, so most of its breeding population is located in one country with a well-kept registry. The contribution of regenerates from other breeds is also quite low. Overestimation of the number of ancestors and founders or underestimation of the inbreeding coefficient is unlikely for such a highly informative pedigree [23].

The analysis of gene origin revealed that the number of founders is extremely low, even compared with that of national breeds with smaller registries such as Polish hounds with 19 [38] or Tatra Shepherd with 55 founders [13]. This is probably caused by the purpose for breed creation. For animals used for laboratory purposes, a high degree of uniformity is desirable, and inbreeding is a classic way to achieve this. Given this fact, there was no need for a large founding population. For the current reference population, there are only nine founders, including three dogs used for regeneration after 1990.

Overused sires and dams were identified on the basis of the limit set in the methodology: 5% of puppies in the last five years. The CSD is a very small breed, and so the limit of maximum descendants per parent was always under 40 puppies since the establishment of the breed up to 2017. In 2001, it reached the limit of 8.1 and thus exceeded for the first time the average size of a litter. According to breeding statistics [39,40,41,42], the average litter size between 2007 and 2017 was approximately 6.3 puppies, and the largest litters did not exceed 11 puppies. The five percent limit was the highest in 2017, identified on the basis of the limit set in the methodology at 37.1 puppies. Based on these facts, the same numbers of puppies for an overused dam or sire were stated individually and separately in each year for 2001–2017. This means that the animals identified this way should not have been used for breeding in a particular year since they have already exceeded the limit counted from the previous five years.

The contribution of founders and ancestors to the reference population was evaluated by the effective number of founders (*f_e_*) and ancestors (*f_a_*). A value of four for *fe* compared to the number of founders shows an uneven contribution of founders. The *f_e_/f* ratio, which would have a value of one with an equal contribution of each founder [28], is only 0.46 for the CSD. Similarly, high values were found mainly for breeds with small registries, such as Skye Terrier 0.49 (Australia), Lakeland Terrier 0.44 (Australia) or the Petite Brabancon 0.50 (Belgium) [8,36].

The ratio between the effective number of ancestors and founders (*f_a_/f_e_*) was used to identify the loss of genetic variability caused both by the bottleneck effect and the uneven use of some individuals for breeding. The effective number of ancestors for the reference population will decrease with the increasing contribution of some founders, to whom a greater number of individuals from the reference population is related to [43]. The effective number of ancestors will therefore decrease and reduce the ratio of *fa* to *f_e_*. For the CSD, the *f_a_/fe* ratio equals 0.75. This means that 75% of genetic losses were caused by the bottleneck effect and uneven founders’ contribution. This is a relatively high value, similar to that observed for Bull Mastiffs registered in Australia [43] or the Italian Pointing Dog in Italy [44]. However, it is necessary to consider how small numbers were used here. Only five ancestors together have a cumulative contribution bigger than 90% in the reference population, in which the two most significant together have more than 50%. This indicates a very uneven use of these animals for breeding. Unfortunately, this trend has been observed in the last 17 years of breeding as well. Altogether, 24 dams and 24 sires with an excessive number of offspring were found in this period, which makes up approximately 8% of all breeding females and 10% of all breeding dogs. In contrast to the proportions reported for the Italian Pointing Dog [44], these numbers represent about ten times more sires and sixteen times more dams. Although the current proportion of overexploited sires and dams in the current population of the CSD is negligible, it is likely to increase in the next generations and further negatively affect genetic parameters of the population. The magnitude of this influence is greater as the population size is smaller, as confirmed by the results of the study on the Polish Hound [38], which shows that only one favored breeding animal suffices for a significant reduction of genetic diversity parameters in the coming years.

Founder genome equivalent (*f_ge_*) also indicates a decrease in genetic variability compared to the original population. This decrease can be understood here as the extent of gene pool diversity of the reference population to its founders [28]. The population studied here carries the same variability extent as 1.31 founders would have. Compared to the Swedish national breeds—most of which have similarly large pedigrees and are strongly endangered by the loss of genetic diversity—the CSD population lost roughly the same proportion of the founding population, approximately 85%. The *f_ge_* values of 12 Swedish national breeds range from 3.31 to 21.57 [45]. However, when averaged and converted to percentages, the loss of diversity compared to the founder population is also more than 80%.

A good indicator of breeding management is the inbreeding coefficient. Though, even between different registries of the same breed, large differences can be observed. For example, in the Nova Scotia Duck Tolling Retriever, the average inbreeding coefficient on a global scale is 0.26 [10], whereas in Australia it is only 0.029, which is almost ten times lower [36]. Although the connection of closely related individuals in most breeder associations is currently prohibited, and the connection of individuals related to the second degree is strongly not recommended, still, mating of distantly related animals is common in practice. The CSD is not an exception [27]. Especially in the very small registry, this is a major problem [25] because over time it is almost impossible to find unrelated individuals, and the inbreeding coefficient is still increasing. This is probably the cause of the high F (0.45) of Bouvier des Ardennes in Belgium [8]. For the CSD, the average inbreeding coefficient for the reference population is 0.365. This is a very high number that could not be obtained even from offspring coming from the union of two own siblings (F = 0.25). At the same time, the average relationship between the individuals of the reference population is more than 75%, and the largest proportion of the reference population (150 out of 233 breeding animals) creates individuals with an inbreeding coefficient of 0.5–0.6.

Additionally, the effective population size, as a measurement of long-term population development as well as a guideline for estimating the maintenance or change in genetic variability in the future, is lower for the CSD compared to that for other breeds. In the Australian Bullmastiff registry, for which the same calculation method for the increase in inbreeding coefficient was used [43], *N_e_* equals 44 individuals, which is four times more than the *N_e_* of the CSD (10.28), even though it is also a breed with a medium registry size. Nearly the same value was obtained for the Italian Pointing Dog (38) [44]. However, the value of *N_e_* below 50 and F above 0.01 indicates a too-rapid loss of heterozygosity, which makes impossible to maintain sufficient genetic variability of the breed in the long term [10].

Based on these facts, it is almost certain that the inbreeding coefficient will continue to increase in the coming years, and that the CSD population will become increasingly homogeneous. Along with this, we can expect a greater incidence of hereditary disorders and diseases currently present, which ceases to have a selected meaning. For the CSD specifically, hip dysplasia, patellar luxation and oligodontia occur [27]. The CSD, therefore, faces the same problem as most of the breeds with small national registries. Because it is a young national breed kept almost exclusively in the Czech Republic, there is no possibility of refreshing blood from abroad. The only possibility remains the infusion of blood of another breed, as this has occurred during the development of the breed several times. However, a one-time outcrossing will only have a short-term effect on the increase in genetic diversity, unless it is regularly repeated [46]. Moreover, descendants of regenerating individuals are phenotypically different from breed standards, which makes them less valuable for kennel judges and breeders [19]. Hard selection and purging the population of deleterious alleles are not suitable for the CSD because of its small registry.

## 5. Conclusions

Many small-registry dog breeds face a shortage of breeding individuals, and this may have consequences in the form of inbreeding depression. The results of this study showed that the CSD breed suffered a serious bottleneck event at the beginning of its existence. In the 1980s, there was a great decline in breeding, which almost led to the extinction of the breed. It was finally restored after 1992. This is also the reason only nine real founders were found in its pedigree. Since that time, records are very complete, which confirms the equivalent complete generation value equal to 10.07. The highly inbred CSD (F = 0.36), as a breed created for uniformity, shows relatively good viability and thus a low frequency of harmful recessive alleles. On the other hand, the CSD population is not completely free of genetic disorders. The breeding methods relatively stabilized the levels of inbreeding, but the uniformity of the population continues to slowly rise. The increase in inbreeding over the last five monitored years was only 1.6%, but breeders did not avoid the excessive use of some animals in breeding. Therefore, a further increase in the incidence of genetic disorders can be expected.

## Figures and Tables

**Table 1:** Development of the Czech Spotted Dog (CSD) population over time.

Period	Mothers	Fathers	Progeny	Registrations	Total Registrations	Founders
**?–1954**	1	1	2	7	7	5
**1955–1960**	1	4	6	6	13	0
**1961–1978**	8	8	19	19	32	0
**1979–1983**	2	2	4	4	36	0
**1984–1985**	3	1	3	3	39	0
**1986–1987**	3	2	6	6	45	0
**1989**	1	1	1	2	47	1
**1990**	0	0	0	1	48	1
**1991**	0	0	0	1	49	1
**1993**	2	2	11	11	60	0
**1994**	2	2	15	16	76	1
**1995**	1	1	7	7	83	0
**1996**	5	3	46	47	130	1
**1997**	1	1	2	2	132	0
**1998**	5	4	28	28	160	0
**1999**	6	5	31	31	191	0
**2000**	10	7	55	55	246	0
**2001**	10	7	59	59	305	0
**2002**	11	8	61	61	366	0
**2003**	11	8	65	65	431	0
**2004**	11	8	71	71	502	0
**2005**	16	14	79	79	581	0
**2006**	13	10	68	68	649	0
**2007**	15	14	71	71	720	0
**2008**	16	12	92	92	812	0
**2009**	20	17	105	105	917	0
**2010**	15	11	94	94	1011	0
**2011**	19	15	119	119	1130	0
**2012**	25	19	160	160	1290	0
**2013**	20	15	125	125	1415	0
**2014**	22	15	137	137	1552	0
**2015**	26	19	155	155	1707	0
**2016**	29	24	165	165	1872	0
**2017**	22	21	138	138	2010	0

**Table 2:** Overused sires and dams and their registration numbers (RNs).

Registration Years	Sires	Dams	Sires RN	Dams RN
**2001**	3	0	292; 333; 358	–
**2002**	4	4	292; 327; 367; 385	269; 305; 306; 362
**2003**	5	4	267; 358; 374; 385; 425	305; 306; 336; 362
**2004**	2	1	374; 358	305
**2005**	2	4	292; 316	336; 362; 405; 477
**2006**	1	3	316	336; 362; 477
**2007**	1	1	267	477
**2008**	0	2	–	449; 477
**2009**	1	1	619	677
**2010**	2	1	409; 619	727
**2011**	1	0	573	–
**2012**	0	1	–	727
**2013**	0	1	–	838
**2014**	1	0	573	–
**2015**	1	1	573	958
**2016**	0	0	–	–
**2017**	0	0	–	–

**Table 3:** Averages of inbreeding (F) and average relatedness (AR) in the pedigree assigned to each generation.

Generation	Number of Animals	AR (%)	F (%)	The Proportion of Inbred Animals (%)	Aver. F of Inbred Animals (%)
**0**	10	10.00	0.00	0.00	0.00
**1**	2	42.32	0.00	0.00	0.00
**2**	2	45.33	12.50	50.00	6.25
**3**	4	60.14	34.38	100.00	34.38
**4**	3	71.56	42.19	100.00	42.19
**5**	6	75.47	47.40	100.00	47.40
**6**	6	77.67	51.46	100.00	51.46
**7**	3	79.43	57.42	100.00	57.42
**8**	3	85.43	55.99	100.00	55.99
**9**	4	89.96	59.21	100.00	59.21
**10**	4	91.82	68.60	100.00	68.60
**11**	9	67.83	30.13	44.44	13.39
**12**	30	65.83	21.98	56.67	12.45
**13**	49	81.17	38.09	75.51	28.76
**14**	104	72.77	32.83	88.46	29.04
**15**	222	72.26	33.02	100.00	33.02
**16**	294	71.51	32.64	100.00	32.64
**17**	337	74.02	35.33	99.70	35.22
**18**	391	74.34	36.00	100.00	36.00
**19**	300	76.80	39.19	100.00	39.19
**20**	193	77.01	39.39	100.00	39.39
**21**	34	77.34	40.04	100.00	40.04

**Table 4:** Distribution of individual inbreeding coefficients in the populations.

Fϵ	In the Whole Population	In the Reference Population
**<0.00; 0.05**	58	2
**<0.15; 0.20**	0	0
**<0.25; 0.30**	82	0
**<0.30; 0.35**	109	9
**<0.35; 0.40**	156	9
**<0.40; 0.45**	335	16
**<0.45; 0.50**	629	38
**<0.50; 0.55**	501	80
**<0.55; 0.60**	78	70
**<0.60; 0.65**	33	9
**<0.65; 0.70**	21	0
